# The Fatty Acid Profile Analysis of *Cyperus laxus* Used for Phytoremediation of Soils from Aged Oil Spill-Impacted Sites Revealed That This Is a C18:3 Plant Species

**DOI:** 10.1371/journal.pone.0140103

**Published:** 2015-10-16

**Authors:** Noemí Araceli Rivera Casado, María del Carmen Montes Horcasitas, Refugio Rodríguez Vázquez, Fernando José Esparza García, Josefina Pérez Vargas, Armando Ariza Castolo, Ronald Ferrera-Cerrato, Octavio Gómez Guzmán, Graciano Calva Calva

**Affiliations:** 1 Biotechnology and Bioengineering, CINVESTAV-IPN, Mexico D. F, México; 2 Biochemical Engineering, Postgraduate Division, TESE, Ecatepec Estado de México, México; 3 Chemistry Department, CINVESTAV-IPN, Mexico D. F., México; 4 Microbiology Area, Edaphology Postgraduate, Postgraduate College, Montecillo, Edo. de México, Mexico; Universidade Federal de Vicosa, BRAZIL

## Abstract

The effect of recalcitrant hydrocarbons on the fatty acid profile from leaf, basal corm, and roots of *Cyperus laxus* plants cultivated in greenhouse phytoremediation systems of soils from aged oil spill-impacted sites containing from 16 to 340 g/Kg total hydrocarbons (THC) was assessed to investigate if this is a C18:3 species and if the hydrocarbon removal during the phytoremediation process has a relationship with the fatty acid profile of this plant. The fatty acid profile was specific to each vegetative organ and was strongly affected by the hydrocarbons level in the impacted sites. Leaf extracts of plants from uncontaminated soil produced palmitic acid (C16), octadecanoic acid (C18:0), unsaturated oleic acids (C18:1-C18:3), and unsaturated eichosanoic (C20:2-C20:3) acids with a noticeable absence of the unsaturated hexadecatrienoic acid (C16:3); this finding demonstrates, for the first time, that *C*. *laxus* is a C18:3 plant. In plants from the phytoremediation systems, the total fatty acid contents in the leaf and the corm were negatively affected by the hydrocarbons presence; however, the effect was positive in root. Interestingly, under contaminated conditions, unusual fatty acids such as odd numbered carbons (C15, C17, C21, and C23) and uncommon unsaturated chains (C20:3n6 and C20:4) were produced together with a remarkable quantity of C22:2 and C24:0 chains in the corm and the leaf. These results demonstrate that weathered hydrocarbons may drastically affect the lipidic composition of *C*. *laxus* at the fatty acid level, suggesting that this species adjusts the cover lipid composition in its vegetative organs, mainly in roots, in response to the weathered hydrocarbon presence and uptake during the phytoremediation process.

## Introduction

Inundation of land with hydrocarbons from oil spills causes long-term contamination of the soil and severe effects on the biodiversity of the ecosystems in the impacted areas [1–3]. The plant community in an oil spill impacted site usually disappears after several months, leaving a large amount of weathered hydrocarbons, such as polycyclic aromatic hydrocarbons (PAH) and asphaltenes [4–8]. The harmful effect of oil spills on the plant community is slowly attenuated by the aging phenomenon, which causes a gradual sequestration of hydrocarbons by soil particles, lowering their accessibility, bioavailability, and biodegradability [9]. After some time, especially in bare areas arising within aged-affected sites, revegetation by the emergence of putative oil-tolerant plant species occurs [5], and it has been hypothesized that revegetation in these oil contaminated sites is a result of phenophases, ecological, and biochemical adjustments of these pioneer plant species to the hydrocarbons’ presence [3,8].

*Cyperus laxus* L. is a member of the Cyperaceae family, which is considered to be a weed cosmopolitan weed located in tropical and subtropical regions [10,11]. It was also recently reported that this and other Cyperaceae species have been identified as pioneer plants in tropical aged and long-term oil spill-impacted sites [12,13]. The ability of this plant species to grow under such stressed conditions may be due that many Cyperaceae species have biochemical traits for using the C4 photosynthetic pathway [14], the C18:3 fatty acid eukaryotic biosynthetic pathway [15], and they also produce underground storage organs such as corms [16]. These characteristics should impart to this plant species greater photosynthetic, biological, and reproductive advantages over other plants to survive in disturbed areas [10]; perhaps for that reason, they are commonly found in both natural disturbed areas [17–19] and anthropogenically disturbed sites [20–22]. Indeed, in similar oil spill-impacted areas previously explored for this work [13], the natural plant community vanished 6–12 months after the oil spill event. Subsequently, in aged or long-term contaminated areas (more than three years), the presence of light oil hydrocarbons was almost negligible, and high amounts of weathered hydrocarbons such as PAH had accumulated. In close sites, Gallegos et al. [12] reported that the average composition of the total hydrocarbon mixture in such areas was: asphaltene (32.4%), aliphatic (39.8%), PAH (18.9%), and polar hydrocarbons (9.1%). Later, we reported that the pioneer plant species found in that aged contaminated areas were *C*. *laxus*, *C*. *esculentus*, *Ludwigia peploides*, *Echinocloa polystachya*, and *Carex crus-corvis*; however, in bare areas emerging from such sites, *C*. *laxus*, *C*. *esculentus*, and *Carex crus-corvis* were the pioneer and dominant species [13]. Interestingly, it has been reported that these Cyperaceae species infest large areas of agricultural lands by producing underground storage organs, such as basal bulbs, corms, and tubers, that enable the regeneration of plants after adverse conditions by acting as perennating organs [23,24]. We have also observed that the Cyperaceae species used for phytotreatment studies of soils from the same areas disturbed by oil spills do produce underground organs [25], and it has also been demonstrated that *C*. *laxus* significantly reduces the hydrocarbon levels from soils containing up to 325,000 mg THC kg^-1^ soil [13,25,26]. Regarding plants cultivated in contaminated soils, during the phytoremediation process, the hydrocarbon removal was associated with changes in the fatty acid composition and the production of unknown conjugated compounds of PAH with some plant metabolites; this suggested that important biochemical adjustments at the fatty acid level in these plants had been accomplished for adapting to the hydrocarbons’ presence. The reason these Cyperaceae species grow in areas disturbed by oil spills and whether there is a relationship between their growth areas and the above cited biochemical characteristics or their production of underground storage organs are unknown. It was hypothesized that the combination of the C18:3 fatty acid biosynthetic pathway with the production of corms might provide *C*. *laxus* with a superior ability to tolerate the weathered hydrocarbons’ toxicity by adjusting the antioxidant capability, thereby producing a higher amount of unsaturated fatty acids in a similar way as reported for the fatty acid content in chicory root cultures grown in presence of benzo(a)pyrene [27]. However, because no information was found about the effect of hydrocarbons on the fatty acid profile of *C*. *laxus* or on the fatty acid profile of other plant species, it is uncertain if *C*. *laxus* uses the potential of the eukaryotic fatty acid pathway to adjust the antioxidant capability against the hydrocarbons in the aged oil spill-impacted sites. Thus, the main objective of this work was to study the changes in the fatty acid profile of *C*. *laxus* plants from the phytoremediation systems to investigate if this is a C18:3 species and if the hydrocarbon removal during the phytoremediation process has a relationship with the fatty acid profile of this plant.

## Materials and Methods

### Phytoremediation systems and plant sampling

**Fig 1:**
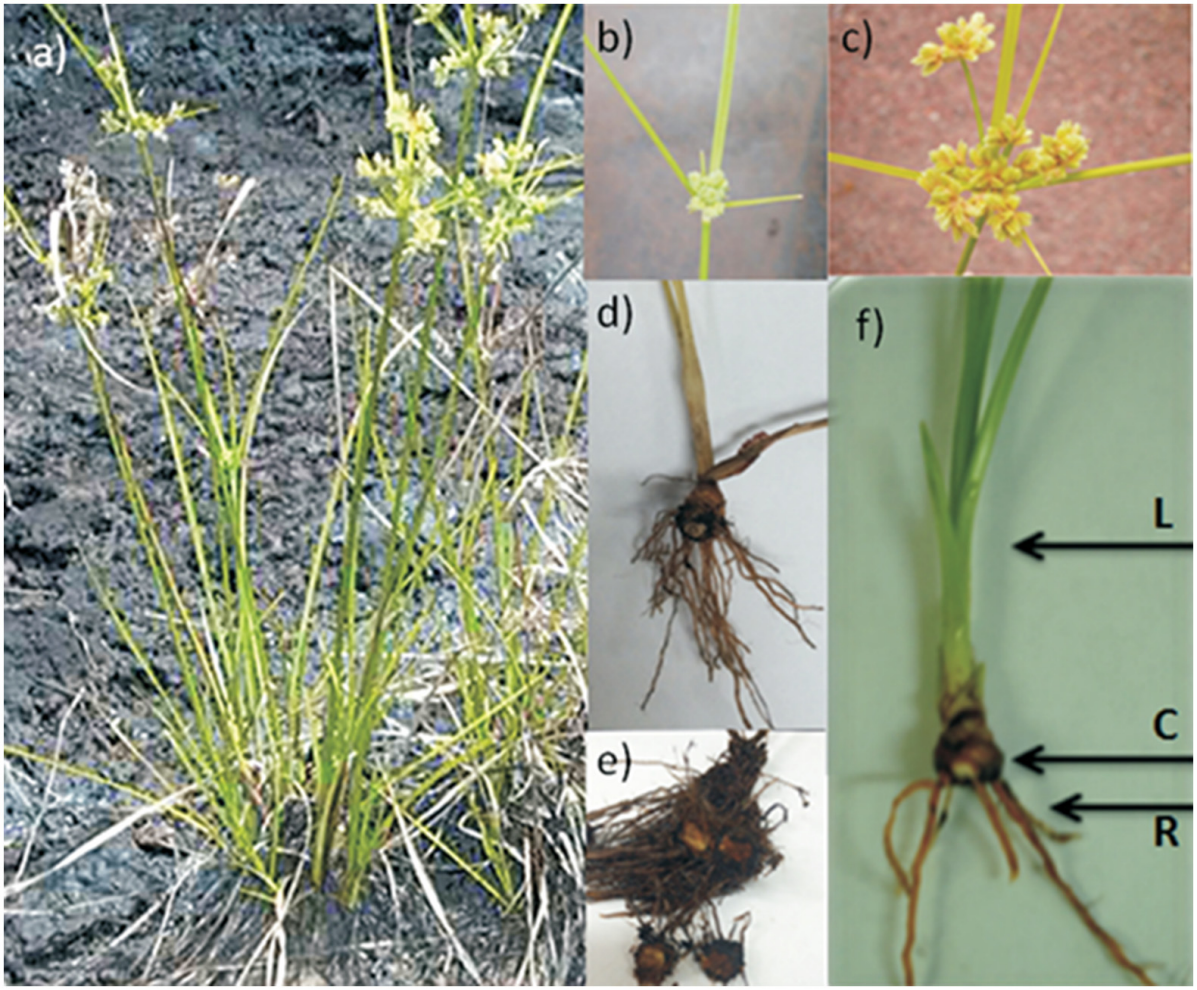
*Cyperus laxus* plants and organs. Pioneer *Cyperus laxus* plants found in aged oil spill-impacted sites located in the tropical region of Tabasco, México (a), their immature (b) and mature (c) inflorescence, and their root system with corms and rhizome (d and e, respectively). Individual plants of 14–15 weeks age from the phytoremediation systems of soil from the aged oil spill-impacted site containing 0–340 g THC/Kg soil were harvested and washed with cold distilled water (f) and the whole leaf (L), the basal corm (C), and the root (R) were separated and used for fatty acid analyses.

Seeds of *Cyperus laxus* were harvested from a three-year greenhouse phytoremediation system established with native plants from long-term oil spill-impacted sites located in the tropical region of Tabasco, México (Fig 1), as previously reported [13,25]. In addition, seeds harvested from plants growing in soil collected from a close unimpacted site (SL) were used for the uncontaminated control system. The seeds were sown in pots (60x20x20 cm) with soil from the control unimpacted site (SL), or with soil from the long-term oil spill-impacted sites containing 16 g/Kg (S163), 140 g/Kg (SSR), and 340 g/Kg (S205) total hydrocarbons (THC), according to a three-stage nested experimental design with five levels of hydrocarbons content and three to five replicates (Table 1). The pot systems were cultivated under greenhouse conditions at 32°C/12°C day/night, and flooded daily with distilled water. Individual plants of 14–15 weeks age from each experimental treatment were harvested and washed with cold distilled water. The whole root, basal corm, and leaf tissue were separated (Fig 1D and 1F). Whole organs from individual plants were then ground under liquid nitrogen for lipid extraction and fatty acid analyses. Additionally, freshly collected plants were used to estimate the humidity content in the organs using a thermobalance (Kern MLB 50–3, Kern & Sohn GmbH, Germany).

**Table 1:** The saturated-unsaturated ratio and the total C12-C24 fatty acid content in the vegetative organs of *Cyperus laxus* cultivated in the phytoremediation systems of soils from oil spill-impacted sites containing several amounts of total hydrocarbons.

	Site and total hydrocarbons content (THC g/Kg Soil) F = 39.6											
F =	SL (0)a 17.3			S163 (16)a 4.9			SSR(140)b 4.7			S205(340)b 19		
ORGAN F = 27.1	Sat/Uns	Uns (%)	Total (mg/g FW)	Sat/Uns	Uns (%)	Total (mg/g FW)	Sat/Uns	Uns (%)	Total (mg/g FW)	Sat/Uns	Uns (%)	Total (mg/g FW)
**LEAF** F = 6.6	1.04	49.10	**1.09±0.02a d**	1.01	49.63	**0.07±0.03c d**	0.95	51.31	**0.51±0.04b d**	0.87	53.55	**0.20±0.02c d**
**CORM** F = 6.5	1.20	45.52	**4.46±0.20a e**	1.20	45.53	**2.98±0.10b e**	2.39	29.48	**1.17±0.06b d**	1.70	37.09	**0.84±0.04c d**
**ROOT** F = 2.5	0.80	55.58	**0.31±0.05a d**	0.50	66.87	**0.55±0.00a d**	0.94	51.67	**1.01±0.02b e**	0.72	58.04	**0.35±0.01a d**

Values of total fatty acid content are the mean ± standard deviation. The F values are the F ratio from the respective ANOVA F-test and using the statistical model FAME-Content = Soil + Organ(soil) + FAME(Organ) +FAME(Organ(Soil)) to estimate the fixed effect by soil and organ. Letters (a, b, c) are groups of equivalent means between soils (Scheffé test, p<0.05), and letters (d, e) below the total fatty acid content are significantly different means between organs (Scheffé test, (p<0.05).

#### Lipid extraction

A modified version of the method reported by Bligh and Dyer [28] for the extraction and purification of lipids from biological materials was performed. The total root (600–800 mg), basal corm (100–300 mg), and leaf (1000–3000 mg) tissue from individual plants were ground in the presence of liquid nitrogen and used to extract the lipidic fraction by vortex mixing (30 seconds) with the proper amount of chloroform-methanol-water (1:2:0.8). This ratio was adjusted according to the humidity content in the tissue (typically 77% for leaf and 51% for corm and root) to have a proportion of 200 mg tissue per mL solvent mixture. Afterward, chloroform (1:1) was added and homogenized by vortex for 20 seconds. Finally, 1 mL of deionized water was added to reach a final proportion of 2:2:1.8 chloroform-methanol-water. The mixture was vortexed for 20 seconds, and the homogenate was filtered to remove the debris. The organic fraction containing the lipids was collected, dried, and resuspended to 1 mL with chloroform.

### Fatty acid methyl esters preparation

The fatty acid profile and composition were determined by preparing the methyl esters of fatty acids (FAME) using a combination of standard procedures reported by Paquot and Hautfenne [29] and Burja et al. [30] for the analysis of oils, fats and derivatives. Samples of 200 μL from the lipid extracts were transferred to 100 mL reaction flasks and evaporated to dryness for saponification by sequential addition of solid sodium hydroxide (2 flakes or 250 mg), water (2 mL), and methanol (20 mL). The reaction mixture was heated (70°C) until it was almost dry, and the unsaponifiable lipids were extracted twice by gentle shaking and decanting with hexane (5 mL). The saponification product was resuspended in hexane (1 mL) and acidified by the addition of 3 mL of concentrated hydrochloric acid (37% p/v) in 20 mL of methanol and the mixture was heated for 2 hours at 70°C for the methylation of the free fatty acids. The mixture was heated until it was almost dry, the volume was adjusted to 20 mL with water, and the FAME were recovered by extraction 3 times with 10 mL of hexane. The organic fraction was concentrated and adjusted to 20 μL with hexane for the gas chromatography (GC) analysis.

### FAME Analysis

The FAME evaluation was performed using a GC and a GC-MS with a mixture of true fatty acid derivatives as reference compounds (SUPELCO 37 Fame Mix, Sigma-Aldrich, México). The experimental conditions were: GC (a 30 m × 0.32 mm HP INNOWAX column; a flame ionization detector with a gradient of 150°C-2´→5°C/min → 200°C-2´→ 260°C; detector 280°C, injection 250°C) and CG-MS (Agilent USC 279167H; 0.50 μm; temperature limit 40°C → 260°C; electronic impact detector).

#### Statistical analysis

A three-stage nested experimental design was used according to a single-fixed factor with four levels of hydrocarbons content and three to five replicates (Table 1). The effect of the fatty acid type (FAME) is nested within the levels of the organ factor, and the effect of the organ factor is nested within the levels of the soil factor [Fatty acid content = Soil + Organ(soil) + FAME(Organ) +FAME(Organ(Soil))]. For the statistical analysis we stated the soil as fixed effect, and the organ and fatty acid type as random effects. The SPSS V 15 statistical package (SPSS Inc., 2006) and the Microsoft Excel 2002 software were used for the statistical analysis and the estimation of the marginal means. The hydrocarbon effect was evaluated using the general linear models (GLM) utility. The post hoc analysis to evaluate the statistical differences between means was performed using the Scheffé test, and the Dunnett test for comparison each of the phytoremediation treatments against the control SL; both at the 0.05 probability level.

## Results and Discussion

### Fatty acid profile in the organs of *Cyperus laxus* cultivated in uncontaminated soil

**Fig 2:**
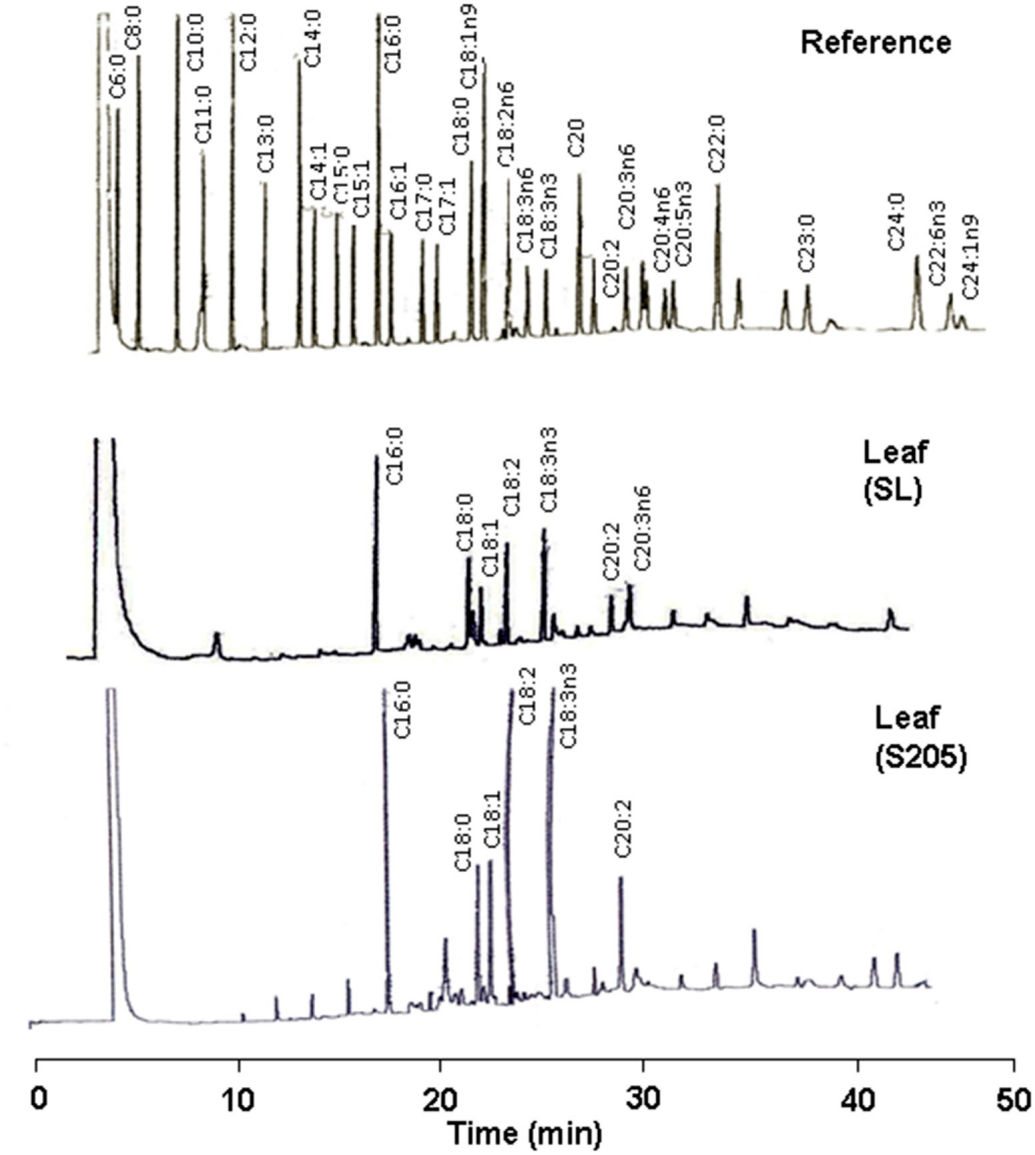
GC Chromatograms of *Cyperus laxus* leaf fatty acid extracts. Fatty acid profile in lipid extracts from the leaf of *Cyperus laxus* control plants cultivated in soil from the unimpacted site (SL) and from plants harvested from the phytoremediation systems of soil from the oil spill-impacted site containing 340 g/Kg THC (S205), compared to a mixture of true fatty acid derivatives (SUPELCO 37 Fame Mix) used as reference compounds.

Fig 2 shows the fatty acid profile of lipid extracts of leaf from plants cultivated in uncontaminated soil (SL) and plants from the phytoremediation system of soil from the oil spill-impacted site containing 340 g/Kg THC (S205) with respect to a mixture of true fatty acids as reference. Approximately 9 fatty acids were clearly identified in the leaf extracts from plants cultivated in the uncontaminated SL with a major occurrence of palmitic acid (C16:0), octadecanoic acid (C18:0), unsaturated oleic acids (C18:1-C18:3), and unsaturated eichosanoic (C20:2-C20:3) acids. The high prevalence of C18:3n3 and the absence of the unsaturated hexadecatrienoic acid (C16:3) in this leaf extract should be noted. These results are consistent with reports for the fatty acid composition of leaf from other Cyperaceae species, such as *Carex* sp. [31] and *Cyperus alternifolius* [15], and demonstrate for the first time that *C*. *laxus* is a C18:3 plant. In C18:3 plants, part of the C16:0, C18:0 and C18:1 fatty acids product of the plastidic prokaryotic fatty acid synthesis are exported to the cytoplasm and incorporated into the endoplasmic reticulum lipids and the polyunsaturated fatty acids through the eukaryotic pathway of glycerolipid synthesis [15,32]. In this work, the presence of hydrocarbons correlated positively with the accumulation of unsaturated fatty acids in leaf (Table 1): the saturated/unsaturated ratio in leaf changed from 1.04 in SL to 0.87 in S205. In contrast, it has been reported that fatty acid desaturation in plants and cyanobacteria are inversely correlated with temperature, and was suggested that such increment in unsaturated fatty acid fraction might improves the fluidity of membrane [32].

The total C12-C24 fatty acid content in the leaf, corm, and root from these SL plants (Table 1), was significantly different (F = 17.3): 1.09, 4.46, and 0.31 mg FA/g FW, respectively. Which, on the basis of a humidity content of 77% for the leaf and 51% for the corm and root, corresponds to 4.8, 9.1, and 0.6 mg FA/g DW, respectively. Because there are no reports for the fatty acid content neither in leaf of *C*. *laxus* nor for the leaf and root of other *Cyperus* species, the results from the present study cannot be compared with data from the literature. However, for corm, the value from this work is in the lower limit of the range reported for tubers or corm from other *Cyperus* species, such as *C*. *rotundus* (6 mg/g DW) or *C*. *esculentus* (51–74 mg /g DW), which usually range from 1 to 300 mg FA/g DW [33–36].

### Fatty acid profile of *Cyperus laxus* from the phytoremediation systems

**Fig 3:**
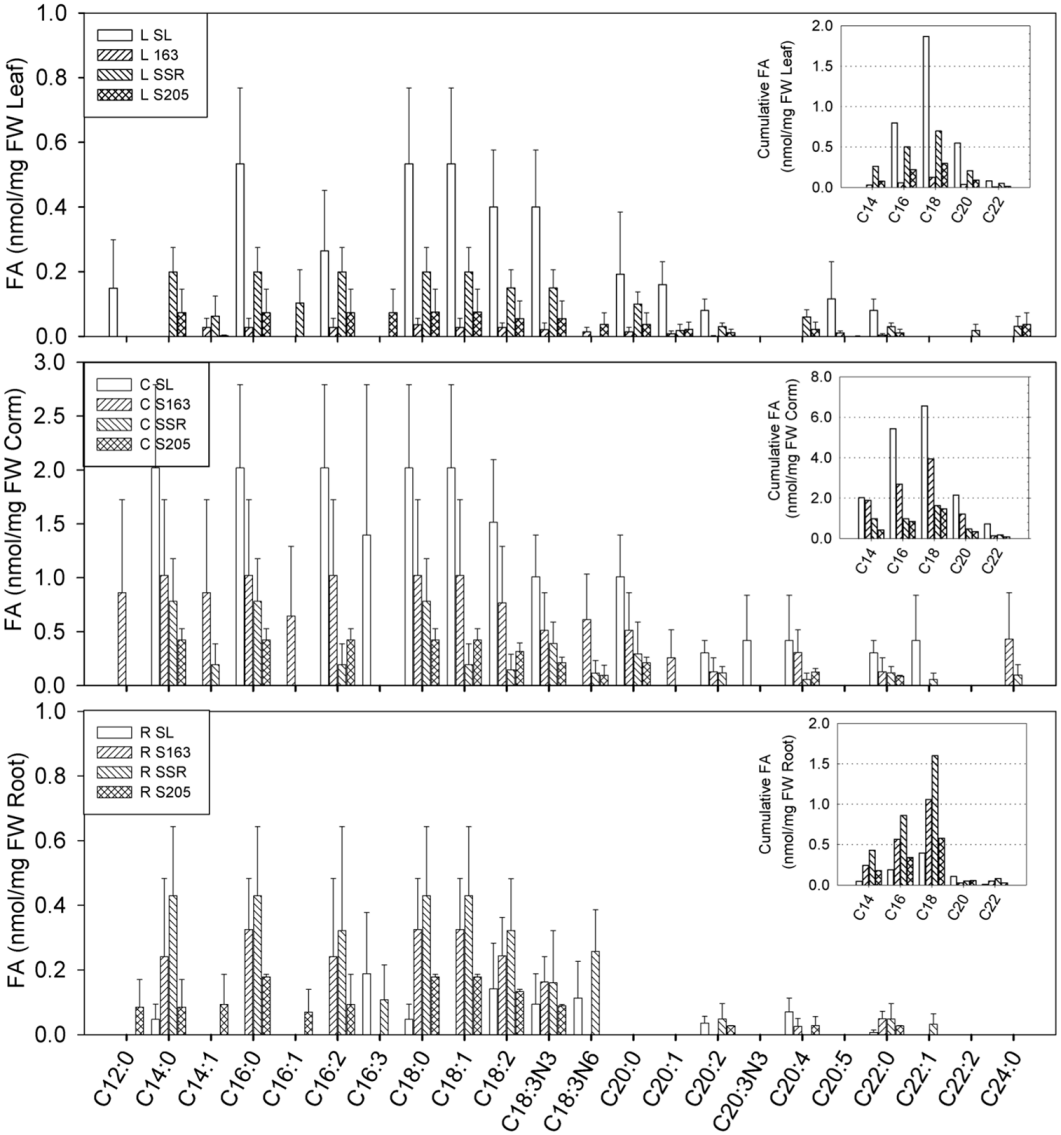
Fatty acid content and distribution in vegetative organs of *Cyperus laxus*. Fatty acid content and fatty acid cumulative chain length distribution (insets) in lipid extracts from leaf, corm, and roots of *Cyperus laxus* plants from the phytoremediation systems of soil from oil spill-impacted sites containing 16 g/Kg (S163), 140 g/Kg (SSR), and 340 g/Kg (S205) of hydrocarbons compared to control plants cultivated in soil from an unimpacted site (SL). Error bars represent the SDS from at least three plants.

**Fig 4:**
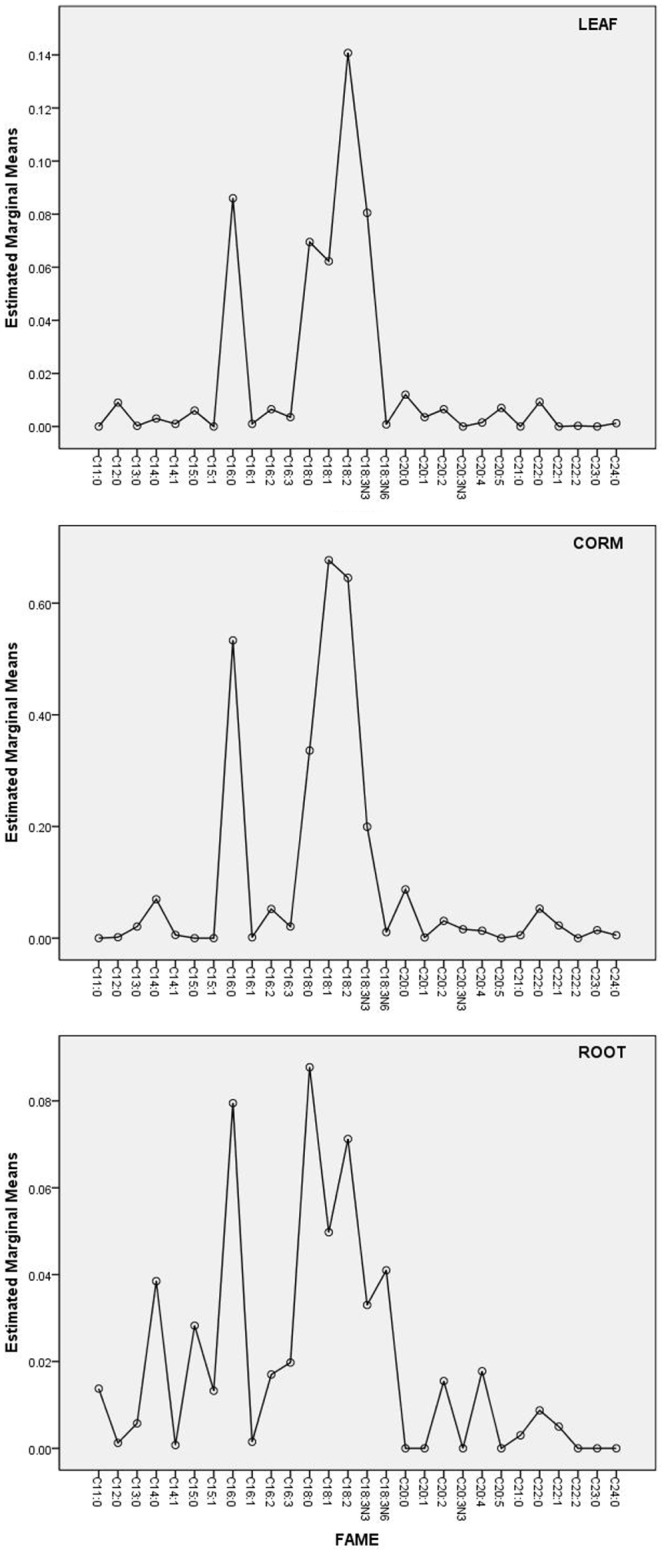
Estimated means for the content of each fatty acid in organs of *Cyperus laxus*. Estimated marginal means for the content of individual fatty acids in leaf, corm, and roots of *Cyperus laxus* based on predictions using the fitted model (Content = Soil + FAME) established from the phytoremediation treatments of soils from the oil spill-impacted sites.

The effect of the THC level on the fatty acid profile (Fig 2), the fatty acid content (Fig 3 and Table 1), and the cumulative chain length distribution (insets in Fig 3) was different for each organ (Fig 4). As shown in the fatty acid profile in the lipid extracts from the leaf of plants from the phytoremediation system of the soil from the impacted site containing 340 g/Kg THC (Fig 2), unusual fatty acids, such as odd numbered carbon (C15, C17, C21, C23) and uncommon unsaturated chains (C20:3n6 and C20:4) were observed together with a remarkable enhancement of the C22:2 and C24:0 chains in the corm and the leaf of the plants from the contaminated soils. The low level or the absence of hexadecatrienoic acid (C16:3) in leaf of plants from the phytoremediation systems (Fig 3), are consistent with the estimated content of C16:3 fatty acid for leaf of plants from the overall phytoremediation treatments of soils from the oil spill-impacted sites based on predictions using the fit model Content = Soil + FAME (Fig 4). This observation agrees with the above results and confirms that *C*. *laxus* is a C18:3 plant.

As shown in Table 1, the total C12-C24 fatty acid content was significantly different between sites (F = 39.6), and between organs for each site (F = 27.1). In plants from the phytoremediation systems, the fatty acid contents in the leaf and the corm were negatively affected by the THC level, but in contrast, for the root tissue, the effect was noticeably positive for a THC content below 340 g/Kg soil. The effect observed for the root tissue is consistent with reports for monoaxenic cultures of *Cichorium intybus* grown in the presence of Benzo[a]pyrene [27] and *Zea mayz* seedlings cultured in the presence of monoterpenes [37], where increases in the total fatty acid content in the roots after the xenobiotic application were observed. However, the results for the corm in the present study contrast with the study of Stoller and Weber [33], where significant increases in the fatty acid content in tubers from a cold tolerant variety of *C*. *esculentus* was reported after a 6-week exposure to a temperature of 2°C.

As expected from the above results, the saturated/unsaturated ratio between organs was positively affected by the hydrocarbon presence (Table 1). For plants from uncontaminated soil (SL, F = 17.), the prevalence of both saturated and unsaturated fractions was equivalent (50%) in the leaf; however, in the root, the unsaturated fraction was predominant (56%) with an absence of C16:2, but with similar amounts of C16:3 and C18:3. In contrast, in the corm, the unsaturated fraction had a noticeably lower prevalence (45%), with a similar content of C16:3 and C18:0-C18:3. These results for corm and leaf from *C*. *laxus* plants grown in uncontaminated soil contrasts with reports of the fatty acid composition of tubers [35, 38,39] and leaves [33] of *C*. *esculentus*, which contain high amounts of monounsaturated fatty acids with a considerably lower prevalence of saturated ones: the saturated/unsaturated rates in the tubers ranged from 0.12 to 0.71, with palmitic acid as the main saturated acid (15%) and oleic acid as the predominant unsaturated acid (72%), and from 0.38 to 0.52 in the leaf, with palmitic acid as the main saturated acid (30%) again, but now with linolenic acid as the predominant unsaturated acid (50%).:

For *C*. *laxus* grown in contaminated soils, as discussed previously, the presence of hydrocarbons correlated positively with the accumulation of unsaturated fatty acids in leaf: the prevalence of the unsaturated fraction changed from 49.1% in SL to 53.6% in S205 (Table 1). However in root the effect was not clear and the unsaturated fraction ranged from 52% to 67% regarding to 55.6% in SL. In contrast, in corm the saturated fraction was prevalent and noticeably enhanced by the hydrocarbon presence: from 54% in the SL control plants to 70% in the plants grown in soil with a THC of 140 g/Kg soil (SSR). As expected, the fatty acid profile was also organ-specific and dramatically affected by the hydrocarbon presence (Fig 3). It is evident that the chain length distribution of the C18 fatty acid group was predominant for each organ (see the insets in Fig 3, and marginal means in Fig 4). However, it should be noted that the major cumulative content of the C18 chain fatty acids was the result of the prevalence of higher and similar amounts of C18:0, C18:1, C18:2, and C18:3N3, in conjunction with low or absent levels of C16:1, C16:2, and C16:3 (Fig 4). For instance, in the leaf from the control plants (SL in Fig 3) the amount and profile of short chain fatty acids (C12-C14) was typically lower than those for mean chain length acids (C16-C18). However, in corm and roots, the amount of C12-C14 was similar to the amount of C16. Interestingly, the presence of hydrocarbons—resulted in an increase in the ratio of short chain fatty acids in the leaf and the roots; however, in the corm a general decrease in these fatty acids was observed. Thus, although the fatty acid profile in each organ was similar between plants cultivated in the same treatment, the presence of hydrocarbons affected the content of some specific fatty acids in the organ. This effect was particularly evident for leaf fatty acids, reducing the content of the C14-C20 group by more than 50%. In contrast, the content of the fatty acids in the roots increased noticeably with the presence of hydrocarbons (Table 1), including the long chain fatty acids C20:2, C22:0 and C22:1, and frequently even in soils containing high levels of THC (Figs 2 and 3). In addition to the typical fatty acids, the presence of small amounts of uncommon uneven and branched carbon chain fatty acids in the roots and the leaf from the phytoremediation systems were detected in the chromatograms (Fig 2), but they were not identified. In summary, the content of fatty acids in the vegetative organs of plants from uncontaminated sites decreased in the order corm>leaf>root; however, for plants grown in soils from contaminated sites, the order was corm>root>leaf (Fig 4).

**Fig 5:**
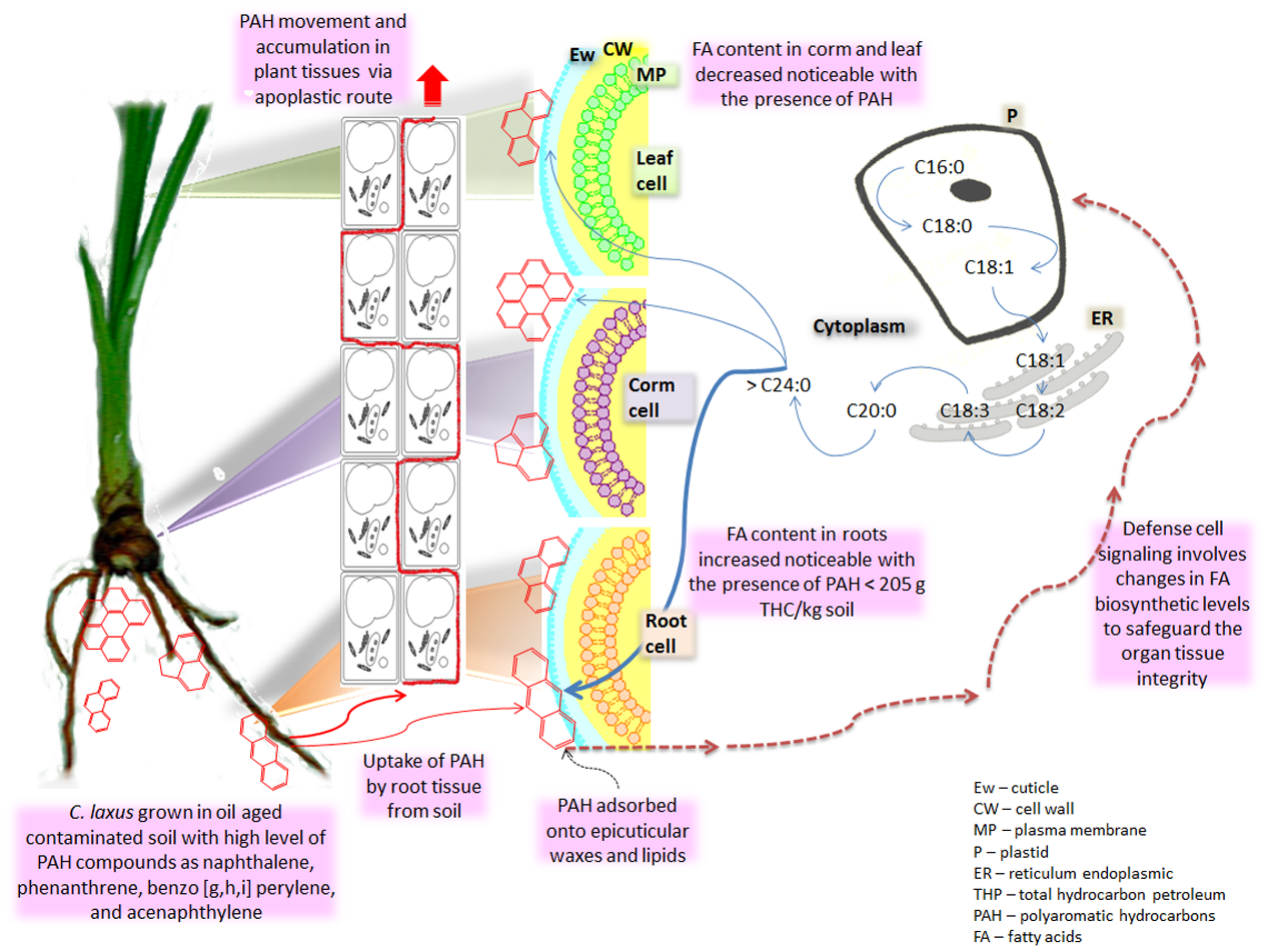
Schematic relationship between PAH and changes in the fatty acid profile of *Cyperus* in the phytoremediation systems. Schematic proposal for the changes observed in the fatty acid profile of organs from *Cyperus laxus* cultivated in the phytoremediation systems of soils from the oil aged contaminated sites. The main type of hydrocarbons found in the aged oil spill impacted sites was PAH^25^. Some of these PAH could be moved through the plant organs by the apoplastic route, promoting a general increase in the content of long chain fatty acids (C18 and C20-C24), mainly in the root; where may negatively affect the translocation process of the micronutrients from the root to the corm and the leaf. In leaf and root tissues the hydrocarbons’ presence did not significantly change the partition of the carbon flux toward any of the fatty acid biosynthetic pathways, keeping both prokaryotic (plastidic) and eukaryotic (ER) fatty acid pathways working in synchrony to maintain a balanced growth. Instead, the major cumulative content of long chain and the C18 chain fatty acids was the result of the prevalence of higher and similar amounts of C18:0, C18:1, C18:2, and C18:3N3, in conjunction with low or absent levels of C16:1, C16:2, and C16:3. This suggest that this *Cyperus* species might direct the intracellular fatty acid metabolic flux to reinforce cellular structures such as plasma membrane (MP), cutine, suberine, and epicuticular wax (Ew), to protect the integrity of the whole plant. Such changes in the fatty acid metabolic flux should involve an important biochemical and physiological adjustment of the plant as response to the hydrocarbons presence. These adjustments may be commonly auto-regulated by metabolic-flexible nodes submerged in the metabolic map of the plant.

As outlined in Fig 5, the above results suggest that in leaf and in root the hydrocarbons’ presence did not significantly affect the partition of the carbon flux toward any of the fatty acid biosynthetic pathways, keeping both prokaryotic and eukaryotic fatty acid pathways working in synchrony to maintain a balanced growth. Therefore, for plants grown in contaminated soil, the increment in the fatty acid content observed in roots in association with a concomitant decrement of total fatty acids in corm and leaf, suggests that the hydrocarbon may have negatively affected the translocation process of the micronutrients from the root to the corm and the leaf, and that the metabolism might have been redirected to the biosynthesis of wax and suberin monomers giving the root cells a higher resistance to the xenobiotic presence. However, the way that the translocation process of the carbohydrates from the photosynthetic tissue to the root tissue was affected is uncertain, and therefore the increase of the fatty acid level in the root by the hydrocarbons’ presence deserves further study. Nevertheless, these results imply that the observed changes in the fatty acids profile were dependent on both hydrocarbons amount and the ability of *C*. *laxus* to adapt the intrinsic cellular lipidic metabolism of each organ in response to the environmental challenge, such as has been reported for other plant species grown under stress conditions [40,41]. Such metabolic adaptation may differentially enhance also the production of uncommon compounds, such as the observation of long chain fatty acids and the presence of branched and uneven carbon chain fatty acids. Long chain fatty acids (C20-C34) are common components or precursors of cellular structures, such as membranes, cuticle, suberine, and waxes [40, 42, 43]. Variations in their profile and content in plants grown in contaminated soils is congruent with reports that suggest that important biochemical and physiological changes at cellular, organ and whole plant levels are involved in the response to the presence of weathered hydrocarbons such as PAH in phytoremediation systems [44–46]. However, because phytoremediation is a very complex system involving interactions between xenobiotics, microbes, plants, and soil, alternative experimental procedures are needed to evaluate the involvement of specific PAH uptake regarding the fatty acid content and profile by plants without microbial interactions.

Why *C*. *laxus* grows in areas disturbed by oil spills, and whether there is a relationship between their growth pattern and the C18:3 biochemical characteristic or their production of underground storage organs is unknown. It is uncertain also whether the increment in the unsaturated fatty acid fraction in the leaf and the root of plants cultivated in the presence of hydrocarbons produce changes in the physical properties of the membrane; however it can be hypothesized that the incorporation of long-chain unsaturated fatty acids to membrane might improve the absorption of such compounds, and therefore their degradation.

Finally, most Cyperaceae species show the Krans anatomy related to C_4_ photosynthesis, and also produce underground storage organs [10,16]; however, it has been reported that *C*. *laxus* lacks the Krans leaf anatomy characteristic of the C4 plants [11, 47], although it does produce corms (Fig 1), and according to its C16:3/C18:3 fatty acid balance in leaf tissue this species is a C18:3 plant. Because some species with C4 photosynthesis do not show a clear Krans anatomy [18, 19], it is uncertain if *C*. *laxus* uses the C4 photosynthetic pathway to grow in the oil-impacted sites, and this subject deserves further research.

## Conclusions

*Cyperus laxus* is a C18:3 plant species that produces corms and is able to survive in soils with high levels of hydrocarbons. The fatty acid profile in the vegetative organs of plants from the phytoremediation systems were noticeably affected by the hydrocarbon levels; showing an increase in the unsaturated fatty acids and the long chain fatty acids in the leaf and root tissue, suggesting that the hydrocarbon uptake during the phytoremediation process depends on the cover lipid composition of the roots. The incorporation of such unsaturated fatty acids in cell membrane of root tissue might improve the absorption and degradation of the hydrocarbon compounds.

## References

[1] Ke L, Teresa WY, Wong YS, Nora FY (2002) Fate of polycyclic aromatic hydrocarbon (PAH) contamination in a mangrove swamp in Hong Kong following an oil spill. Mar Pollut Bull 45: 339–347. 12398405 10.1016/s0025-326x(02)00117-0

[2] Lin Q, Mendelssohn IA, Carney K, Bryner NP, Walton WD (2002) Salt marsh recovery and oil spill remediation after in-situ burning: Effect of water depth and burn duration. Environ Sci Technol 36: 576–581. 11878369 10.1021/es011075l

[3] Lawer EA, Baatuuwie BN, Ochire-Boadu K, Jasper Asante W (2013) Preliminary assessment of the effects of anthropogenic activities on vegetation cover and natural regeneration in a moist semi-deciduous forest of Ghana. Int J Ecosys 3: 148–156.

[4] Gentry AH (1988) Changes in plant community diversity and floristic composition on environmental and geographical gradients. Ann Mo Bot Gard 75: 1–34.

[5] Mendelssohn IA, Lin Q, Bryner NP, Walton WD, Twilley WH, Mullin JV (2002) In-Situ oil burning in the marshland environment- recovery and regrowth of Spartina alterniflora, Spartina patens, and Sagittaria lancifolia plants. In: Proceedings of the Twenty-Fifth artic and Marine Oil Spill Program Technical Seminar: Environment Canada. pp. 785–802. Available: http://fire.nist.gov/bfrlpubs/fire02/PDF/f02068.pdf.

[6] Lot A (2004) Flora y vegetación de los humedales de agua dulce en la zona costera del Golfo de México In: Caso M, Pisanty I, Ezcurra E, editors. Diagnóstico ambiental del Golfo de México: Instituto Nacional de Ecología, México pp. 521–553.

[7] Merkl N, Schultze-Kraft R, Infante C (2004) Phytoremediation of petroleum-contaminated soils in the tropics—preliminary assessment of the potential of species from eastern Venezuela. J Appl Bot Food Qual 78: 185–192.

[8] Lin Q, Mendelssohn IA, Bryner NP, Walton WD (2005) In-situ burning of oil in coastal marshes. 1: Vegetation recovery and soil temperature as a function of water depth, oil type and marsh type. Environ Sci Technol 39: 1848–1854. 15819246 10.1021/es049063y

[9] Semple KT, Doick KJ, Jones KC, Burauel P, Craven A, et al (2004) Defining bioavailability and bioaccessibility of contaminated soil and sediment is complicated. Environ Sci Technol 38: 228A–231A. 15260315 10.1021/es040548w

[10] Bryson CT, Carter R (2008) The significance of Cyperaceae as weeds In: Naczi RF, Ford BA, editors. Sedges, uses, diversity, and systematic of the *Cyperaceae*. Missouri: Monographs in Systematic Botany from the Missoure Botanical Garden pp. 15–101.

[11] Martins S, Scatena VL (2013) Developmental anatomy of Cyperus laxus (non-Nranz) and Fimbristylis dichotoma (Kranz) (Cyperaceae, Poales) and tissue continuity. An Acad Bras Ciênc 85: 605–613. 10.1590/S0001-37652013005000032 23828350

[12] Gallegos Martínez M, Gomez Santos A, González Cruz L, Montes de Oca García MA, Yañez Trujillo L, Zermeño Eguía Lis JA, et al (2000) Diagnostic and resulting approaches to restore petroleum contaminated soil in a Mexican tropical swamp. Water Sci Technol 42: 377–384.

[13] Palma CFJ, Esparza García FJ, Poggi Varaldo HM, Rodríguez Vázquez R, Peña Cabriales JJ, Ferrera- Cerrato R, et al. (2004) Changes in the number of plant species in sites from Tabasco, México, chronically polluted with oil. In: México DF, México CD, editors. The First International Meeting on Environmental Biotechnology and Engineering. Institut de recherche pour le developpement/CINVESTAV. Paper IMEBE 152.

[14] Voznesenskaya EV, Franceschi VR, Kiirats O, Freitag H, Edwards GE (2001) Kranz anatomy is not essential for terrestrial C4 plant photosynthesis. Nature 414: 543–546. 11734854 10.1038/35107073

[15] Mongrand S, Bessoule JJ, Cabantous F, Cassagne C (1998) The C16:3/C18:3 fatty acid balance in photosynthetic tissue from 468 plant species. Phytochemistry 38: 1049–1064.

[16] Dominy NJ, Vogel ER, Yeakel JD, Constantino P, Lucas PW (2008) Mechanical properties of plant underground storage organs and implications for dietary models of early hominins. Evol Biol 35: 159–175: 10.1007/s11692-008-9026-7

[17] Ueno O, Takeda T (1992) Photosynthetic pathways, ecological characteristics, and the geographical distribution of the Cyperaceae in Japan. Oecologia 89: 195–203. 28312873 10.1007/BF00317218

[18] Martins S, Alves M (2009) Anatomical features of species of Cyperaceae from northeastern Brazil. Brittonia 61: 189–200.

[19] Lundgren MR, Osborne CP, Christin P (2014) Deconstructing Kranz anatomy to understand C4 evolution. J Exp Bot 65 (Special Issue): 3357–3369. 24799561 10.1093/jxb/eru186

[20] Lovera M, Cuenca G (1996) Arbuscular mycorrhizal infection in Cyperaceae and Gramineae from natural, disturbed and restored savannas in la Gran Sabana, Venezuela. Mycorrhiza 6: 111–118.

[21] Deng H, Ye ZH, Wong MH (2004) Accumulation of lead, zinc, copper and cadmium by 12 wetland plant species thriving in metal-contaminated sites in China. Environ Pollut 132: 29–40. 15276271 10.1016/j.envpol.2004.03.030

[22] García-López E, Zavala-Cruz J, Palma-López DJ (2006) Caracterización de las comunidades vegetales en un área afectada por derrames de hidrocarburos. Terra (Latinoamericana) 24: 17–26.

[23] Daehler CC (1998) The taxonomic distribution of invasive angiosperm plants: Ecological insights and comparision to agricultural weeds. Biol Conserv 84: 167–180.

[24] Eyherabide JJ, Leaden MI, Alonso S (2001) Yellow and purple nutsedges survey in the southeastern Buenos Aires Province, Argentina. Pesq Agropec Bras 36: 205–209.

[25] Rivera-Casado NA, Montes-Horcasitas MC, Esparza-Garcia FJ, Ariza-Castolo A, Gómez-Guzmán O, Pérez Vargas J, et al (2010) Phytotreatment of oil spill impacted soils: interaction between polyaromatic hydrocarbons, phenolics and oxidative enzymes. Revista CENIC Chemical Sciences 41:1–11. Available: http://www.redalyc.org/articulo.oa?id=181620500028. Accesed 27 november 2014.

[26] Rivera Casado NA, Montes Horcasitas MC, Rodríguez Vázquez R, Esparza García FJ, Ariza Castolo A, Gómez Guzmán O, et al (2013) Changes in fatty acid composition of C. *L*axus in a phytotreatment system of petroleum-contaminated soil In: Sirabian RR, Darlington R, editors. Second International Symposium on Bioremediation and Sustainable Environmental Technologies. Columbus, OH: Battelle Memorial Institute pp. A–72. Available at: www.battelle.org/biosymp.

[27] Debiane D, Calonne M, Fontaine J, Laruelle F, Grandmougin-Ferjani A, Lounès-Hadj Sahraoui A (2012) Benzo[a]pyrene induced lipid changes in the monoxenic arbuscular mycorrhizal chicory roots. J Hazard Mater 209: 18–26. 22277337 10.1016/j.jhazmat.2011.12.044

[28] Bligh EG, Dyer WJ (1959) A rapid method for total lipid extraction and purification. Can J Biochem Physiol 37: 911–917. 13671378 10.1139/o59-099

[29] Paquot C, Hautfenne A (1987) Standard methods for the analysis of oils, fats, and derivatives. Oxford: Blackwell Scientific Publications.

[30] Burja AM, Armenta RE, Radianingtyas H, Barrow CJ (2007) Evaluation of fatty Acid extraction methods for Thraustochytrium sp. ONC-T18. J Agric Food Chem 55: 4795–4801. 17497884 10.1021/jf070412s

[31] Bogucka-Kocka A, Janyszek M (2010) Fatty acids composition of fruits of selected Central European sedges, *carex L*. (*Cyperaceae*). Grasas Aceites 61: 165–170.

[32] Somerville C, Browse J, Jaworski JG, Ohrologge JB (2000) Lipids In: Buchanan B, Gruissem W, Jones R, editors. Biochemistry and Molecular Biology of Plants: Rockville, MD: American Society of Plant Biologists pp. 456–527.

[33] Stoller EW, Weber EJ (1975) Differential Cold tolerance, starch, sugar, protein, and lipid of yellow and purple nutsedge tubers. Plant Physiol 55: 859–863. 16659181 10.1104/pp.55.5.859 PMC541723

[34] Matthiesen RL (1976) Plant development and tuber composition of six biotypes of yellow nutsedge Cyperus esculentus L. PhD Thesis. University of Illinoids at Urbana-Champaign. p. 86. Available: http://eurekamag.com/research/000/460/000460157.php. Accessed 5 September 2014.

[35] Turesson H, Marttila S, Gustavsson KE, Hofvander P, Olsson ME, Bülow L, et al (2010) Characterization of oil and starch accumulation in tubers of Cyperus esculentus var. sativus (Cyperaceae): A novel model system to study oil reserves in nonseed tissues. Am J Bot 97: 1884–1893. 10.3732/ajb.1000200 21616827

[36] Ezeh O, Gordon MH, Niranjan K (2014) Tiger nut oil (Cyperus esculentus L.): a review of its composition and physico‐chemical properties. Eur J Lipid Sci Technol 116: 783–794.

[37] Zunino MP, Zygadlo JA (2005) Changes in the composition of phospholipid fatty acids and sterols of maize root in response to monoterpenes. J Chem Ecol 31: 1269–1283. 16229065 10.1007/s10886-005-5285-2

[38] Arafat SM, Gaafar AM, Basunu AM, Nassef SL (2009) Chufa tuber (Cyperus esculentus L.): a new source of food. World Appl Sci J 7: 151–156.

[39] Lopéz-Cortés I, Salazar-García DC, Malheiro R, Guardiola V, Pereira JA (2013) Chemometrics as a tool to discriminate geographical origin of Cyperus esculentus L. Based on chemical composition. Ind Crops Prod 51: 19–25.

[40] Kolattukudy PE (1970) Biosynthesis of cuticular lipids. Annu Rev Plant Physiol 21: 163–192.

[41] Kroumova AB, Xie Z, Wagner GJ (1994) A pathway for the biosynthesis of straight and branched, odd- and even-lenght, medium-chain fatty acids in plants. Proc Natl Acad Sci U. S. A. 91: 11437–11441. 7972080 10.1073/pnas.91.24.11437 PMC45246

[42] Lessire R, Hartmann-Bouillon M, Cassagne C (1982) Very long chain fatty acids: Occurrence and biosynthesis in membrane fractions from etiolated maize coleptiles. Phytochemistry 21: 55–59.

[43] Kunst L, Samuels AL (2003) Biosynthesis and secretion of plant cuticular wax. Prog Lipid Res 42: 51–80. 12467640 10.1016/s0163-7827(02)00045-0

[44] Bell RM, Coleman RA (1980) Enzymes of Glycerolipid synthesis in eukaryotes. Annu Rev Biochem 49: 459–487. 6250446 10.1146/annurev.bi.49.070180.002331

[45] Malallah G, Afzal M, Kurian M, Gulshan S, Dhami MSI (1998) Impact of oil pollution on some desert plants. Environ Int 24: 919–924.

[46] Peña-Castro JM, Barrera-Figueroa BE, Fernández-Linares L, Ruiz-Medrano R, Xoconostle-Cázares B (2006) Isolation and identification of up-regulated genes in Bermudagrass roots (Cynodon dactylon L.) grown under petroleum hydrocarbon stress. Plant Science 170: 724–731.

[47] Bruhl JJ, Wilson KL (2007) Towards a comprehensive survey of C3 and C4 photosynthetic pathways in Cyperaceae. Aliso: A Journal of Systematic and Evolutionary Botany. 23: 99–148. Available: http://scholarship.claremont.edu/aliso/vol23/iss1/11. Accessed 20 September 2014.

